# Supplementary material for: Lateral Diffusion of NKCC1 Contributes to Chloride Homeostasis in Neurons and Is Rapidly Regulated by the WNK Signaling Pathway
Source: Cells. 2023 Jan 31;12(3):464. doi: 10.3390/cells12030464 (PMC9914440; doi:10.3390/cells12030464)
Supplement: Supplementary file 1 [file cells-12-00464-s001.zip › cells-2120207-supplementary.pdf]

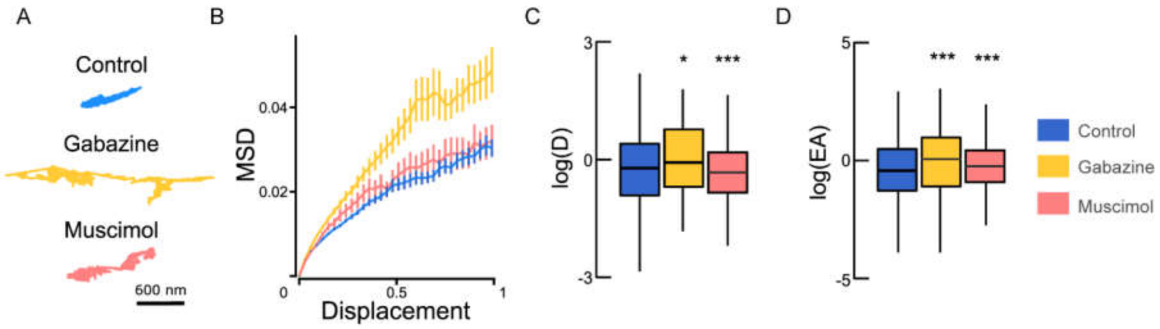

Figure S1. NKCC1 diffusion in the axon is increased upon GABAAR blockade: **A,** Reconstructed trajectories of NKCC1 in the axon depicting the increased surface exploration upon gabazine (yellow) and muscimol (orange) treatment as compared to control (blue). **B,** Time-averaged MSD functions of axonal QDs in control (blue) vs. gabazine (yellow) or muscimol (orange) exposure. The MSD versus time relationship for axonal trajectories shows a steeper initial slope upon drug exposure, suggesting that trajectories were less confined. $N_{Control} = 390$ QDs, $N_{gabazine} = 145$ QDs, $N_{muscimol} = 274$ QDs, 37 cells from 5 cultures. **C-D**, Boxplots of log(D) of NKCC1 in control condition (blue) or upon application of gabazine (yellow) or muscimol (orange) showing increased diffusion upon gabazine application. N = 390 QDs (control, 48 cells), n = 145 QDs (gabazine, 64 cells), Welch t-test, $p = 3.5 \times 10^{-2}$, n = 274 QDs (muscimol, 28 cells), Welch t-test, $p = 2.1 \times 10^{-12}$, 5 cultures. **D**, Median explored area EA in control vs. gabazine or muscimol conditions show reduced confinement upon gabazine (Welch t-test, $p = 8.46 \times 10^{-6}$) or muscimol (Welch t-test, $p = 4.2 \times 10^{-11}$) application. C: D in $\mu m^2.s^{-1}$; D: EA in μm^2. In all graphs, *, $p < 5.0 \times 10^{-2}$; ***, $p < 1.0 \times 10^{-3}$.
